# Maternal internalizing symptoms as a mechanism linking pre- and postnatal COVID-19 pandemic exposure with preschool-aged children’s neurodevelopment

**DOI:** 10.1007/s00737-026-01686-2

**Published:** 2026-03-26

**Authors:** Katherine E. Finegold, Julia A. Knight, Rayjean J. Hung, Cindy-Lee Dennis, Prakesh S. Shah, Jody Wong, Kashtin Bertoni, Robert D. Levitan, Jennifer M. Jenkins, Stephen G. Matthews, Mark Wade

**Affiliations:** 1 Department of Applied Psychology and Human Development, University of Toronto, 252 Bloor Street West, Toronto, ON Canada M5S1V6; 2 Prosserman Centre for Health Research, Lunenfeld-Tanenbaum Research Institute, Sinai Health, Toronto, Canada; 3 Division of Epidemiology, Dalla Lana School of Public Health, University of Toronto, Toronto, Canada; 4 Lawrence S. Bloomberg Faculty of Nursing, University of Toronto, Toronto, Canada; 5 Lunenfeld-Tannenbaum Research Institute, Mount Sinai Hospital, Toronto, Canada; 6 Department of Paediatrics, University of Toronto, Toronto, Canada; 7 Department of Paediatrics, Sinai Health, Toronto, Canada; 8 Mood and Anxiety Disorders Program, Centre for Addiction and Mental Health, Toronto, Canada; 9 Department of Psychiatry, University of Toronto, Toronto, Canada; 10 Department of Obstetrics and Gynaecology, Sinai Health System, Toronto, Canada; 11 Department of Obstetrics and Gynaecology, University of Toronto, Toronto, Canada

**Keywords:** Ontario birth study, COVID-19 pandemic, Maternal anxiety, Maternal depression, Prenatal mental health, Early childhood neurodevelopment

## Abstract

**Purpose:**

There is some evidence that children exposed to the COVID-19 pandemic experienced more neurodevelopmental difficulties than children before the pandemic, as well as evidence that women with young children experienced more mental health challenges during this period compared to pre-pandemic. However, it is unclear whether increased maternal mental health challenges acted as a mechanism linking pandemic exposure to children’s neurodevelopment difficulties.

**Methods:**

As part of the Ontario Birth Study, women (*N* = 862) reported their internalizing (i.e., depression and anxiety) symptoms using the Patient Health Questionnaire-4 (PHQ-4) at three timepoints (prenatally and 8 and 24 months postnatally). Child neurodevelopment was assessed at 24 months using the Ages and Stages Questionnaire-3 (ASQ-3). Analyses included a combination of regression and path analyses with adjustment for covariates.

**Results:**

Women exposed to the pandemic prenatally and at 8 and 24 months postnatally reported more concurrent internalizing difficulties than those not exposed; however, women exposed both pre- and postnatally did not differ from those only exposed postnatally. Higher prenatal maternal internalizing symptoms were associated with lower child gross motor skills at 24 months. Higher maternal internalizing symptoms at 8 and 24 months were marginally (*p* < 0.1) associated with lower child personal-social and gross motor skills, respectively, at 24 months. The association between prenatal pandemic exposure and lower gross motor skills was marginally mediated by the presence of prenatal maternal internalizing symptoms.

**Conclusion:**

Mothers and young children may have been particularly vulnerable to pandemic stress. Maternal internalizing symptoms, especially during pregnancy, may serve as a pathway linking pandemic exposure with child neurodevelopment and may represent a malleable target for intervention.

**Supplementary Information:**

The online version contains supplementary material available at 10.1007/s00737-026-01686-2.

## Key Highlights

- Women exposed to the pandemic both prenatally and postnatally had greater internalizing difficulties compared to non-exposed mothers.
- Higher maternal internalizing symptoms prenatally was associated with lower child gross motor skills at 24 months.
- Higher maternal internalizing symptoms at 8 and 24 months were marginally associated with lower child personal-social and gross motor skills, respectively, at 24 months.
- The presence of any maternal internalizing symptoms marginally mediated the association between prenatal pandemic exposure and children’s gross motor abilities.

**Supplementary Information:**

The online version contains supplementary material available at 10.1007/s00737-026-01686-2.

## Introduction

The COVID-19 pandemic was associated with significant social disruptions that impacted family functioning and well-being (Prime et al. 2020). Most studies examining the impact of the pandemic have focused on school-age youth, with fewer centered on the well-being of preschool-aged children (Viner et al. 2022). Among studies including young children, most have explored the effects of the pandemic on socioemotional, rather than neurodevelopmental, outcomes (Alcon et al. 2024; Jing et al. 2024; Kuehn et al. 2024). Preschool-aged children may have been especially vulnerable to the pandemic due to the brain’s heightened sensitivity to stress early in development, emphasizing the importance of understanding how the pandemic may have affected this age group across different domains of development (Nelson & Gabard-Durnam 2020). Studies exploring the association between the pandemic and preschool-aged children’s neurodevelopment have yielded mixed results, with some studies suggesting lower communication, gross motor, fine motor, and personal-social skills among pandemic-exposed, compared to non-exposed, children (Finegold et al. 2023; Huang et al. 2021; Shuffrey et al. 2021). However, studies have also shown no differences, or even higher scores, among pandemic-exposed children in domains such as problem-solving and fine motor skills (Byrne et al. 2023; Finegold et al. 2023). Still, the mechanisms explaining why pandemic exposure is related to children’s neurodevelopmental outcomes remain poorly understood (Firestein & Shuffrey 2023). It is also unclear whether the timing of pandemic exposure (e.g., prenatally vs. postnatally) may have been differentially associated with child outcomes.

Maternal mental health is one potential pathway linking pandemic exposure to children’s developmental outcomes. Women are more susceptible to mental health challenges during the first postpartum year (Shorey et al. 2018), and this vulnerability may have been exacerbated by pandemic stress. At the beginning of the pandemic, mothers of young children were at an increased risk for mental health problems (Pierce et al. 2020), with increased rates of anxiety, depression, and distress compared to pre-pandemic levels (Cameron et al. 2020; Racine et al. 2021). While maternal depression and anxiety are associated with behavioral, emotional, and cognitive outcomes in children (Leijdesdorff et al. 2017), it remains unclear whether maternal mental health problems mediate the link between pandemic exposure and children’s outcomes.

Most evidence regarding children’s development during the pandemic is based on postnatal exposure to the pandemic. Few studies have explored whether prenatal pandemic exposure is longitudinally associated with child outcomes. Indeed, prenatal stress impacts child development through alterations in biological processes in utero such as epigenetic changes and exposure to glucocorticoids (Cao-Lei et al. 2020; Van den Bergh et al. 2020). In turn, increased stress and more mental health symptoms during pregnancy are linked to neurophysiological, psychological, and developmental challenges in children (Chan et al. 2018; Delagneau et al. 2023; Kingston et al. 2015; Lebel et al. 2016; Madigan et al. 2018). During the pandemic, pregnant women reported high levels of anxiety, depression, negative affect, and posttraumatic stress symptoms (Berthelot et al. 2020; Tomfohr-Madsen et al. 2021). However, the unique risks posed by prenatal pandemic exposure for maternal mental health and child neurodevelopment are underexplored.

The present study used data from a longitudinal birth cohort that began before and continued during the pandemic. We examined associations between pandemic exposure (compared to non-exposure) with maternal internalizing symptoms and child neurodevelopment up to 24 months of age, as well as whether maternal internalizing difficulties mediated potential effects of pandemic exposure pre- and postnatally on children’s development.

## Methods

### Study design and participants

Participants were from the Ontario Birth Study (OBS), a large cohort study initiated in 2013 that continued recruitment through the pandemic. Women who were receiving prenatal care at Mount Sinai Hospital in Toronto, Ontario and were less than 17 weeks gestation were eligible to participate in the study (Anderson et al. 2018). During pregnancy, participants completed online questionnaires, which included measures of maternal mental health and sociodemographic factors at 12–16 weeks and 28–32 weeks gestation. A companion protocol, the OBS Kids, was established in 2018 to follow OBS mothers and their children through early childhood. Informed consent was obtained for all women who agreed to participate in OBS and OBS Kids (Anderson et al. 2018). Participants completed questionnaires when their child turned 8 months and had home visits at 24 months to measure children’s development. Participants who had completed the 24-month assessment (*N* = 862) were included in the present study. Twenty-four twins were excluded (one twin of each twin pair was selected using a random number generator), leaving 838 participants for analyses. Participants in this study completed assessments between July 15, 2015 and February 1, 2024. This study was approved by the research ethics boards at the University of Toronto (REB #45278) and Sinai Health (REB #636). Sinai Health approved data collection by the OBS (REB #1164) and OBS Kids (REB #1024).

### Measures

#### Pandemic exposure

At each timepoint (prenatal, 8 months, and 24 months), participants were considered part of the *non-exposed* group if their assessment for that specific timepoint occurred before March 11, 2020. Participants were considered part of the *pandemic-exposed* group if their assessment for that specific timepoint was on or after March 11, 2020 through the study end date, February 1, 2024. For most participants, their assessment occurred between March 11, 2020 and May 5, 2023, the date the WHO declared the end of the pandemic. However, 32 participants completed their 24-month assessment between May 5, 2023 and the study end date of February 1, 2024. These participants are included as part of the pandemic-exposed group because they were exposed to the pandemic prenatally and for a significant part of their postnatal life. Sensitivity analyses were conducted to determine if observed associations held once excluding those assessed after May 5, 2023 (Tables S1 and S2).

**Fig. 1:**
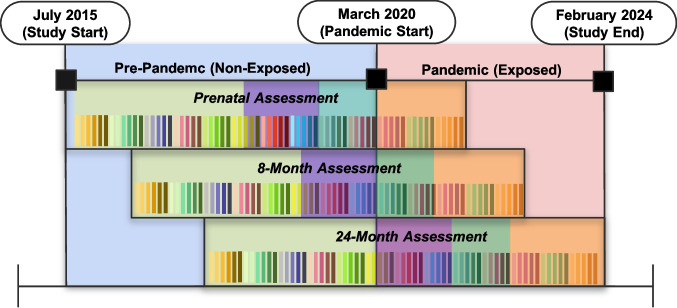
Timeline depicting the different COVID-19 pandemic exposure groups based on when participants were assessed as part of the study. The timeline is divided into two main segments: (i) pre-pandemic period (July 2015 to when the pandemic began in March 2020), in which participants are classified as “non-exposed” for that specific timepoint if their assessment occurred during that window (blue shaded area on the timeline); and (ii) pandemic period (March 11, 2020 onward), in which participants are classified as “exposed” for that specific timepoint if their assessment occurred during that window (red shaded area). Each colored vertical line represents a unique participant who was assessed at each timepoint. Recruitment for the study was ongoing, and participants completed each assessment (prenatal, 8 months, and 24 months) when they reached the age of the assessment. This means some participants may have completed all assessment pre-pandemic (yellow shaded area), some may have been non-exposed prenatally and at 8 months postnatally but exposed at 24 months postnatally only (purple shaded area), some may have been non-exposed prenatally but exposed at both 8 and 24 months postnatally (green shaded area), and some may have been exposed at all three timepoints (orange shaded area). Numbers of participants represented in each group are not proportional to actual study numbers, and are only meant for illustrative purposes (see in-text for exact numbers in each group)

Within the *pandemic-exposed* group, participants were further categorized as: (1) *prenatal-exposed* if their assessment took place during at least one pregnancy timepoint during the pandemic; (2) *postnatal-only exposed* if their assessment took place only after birth during the pandemic; and (3) as *pre/postnatal-exposed* if their assessment took place prenatally during the pandemic and during at least some postnatal timepoints—since all children exposed to the pandemic prenatally were also exposed once they were born, we call this the *pre/postnatal-exposed* group (see Fig. 1 for depiction of the study design). Mothers and their children were considered participants in the study.

Comparisons between the following groups were made: (1) non-exposed vs. postnatal-only exposed; (2) non-exposed vs. pre/postnatal-exposed; and (3) postnatal-only exposed vs. pre/postnatal-exposed. At 8 and 24 months, postnatal-only exposure was defined as having one’s assessment take place at that specific age during the pandemic. Given the ongoing enrollment design, some mothers and children completed both the 8- and 24-month assessments during the pandemic, while others completed only the 24-month evaluation during this period. We treated these groups separately to examine the cumulative effect of pandemic exposure on child development.

#### Maternal internalizing symptoms

Maternal internalizing symptoms were assessed at each timepoint (prenatally at 12–16 and 28–32 weeks gestation and postnatally at 8 and 24 months) using the Patient Health Questionnaire- 4 (PHQ-4), a well-validated self-report measure of anxiety and depression symptoms (Kroenke et al. 2009). Participants rated items on a 4-point scale from 0 (“not at all”) to 3 (“nearly every day”). Total scores, ranging from 0 to 12, were used in the analyses. For prenatal internalizing problems, we took an average of the total scores at 12–16 weeks and 28–32 weeks gestation. The internal consistency of the PHQ-4 at each pre- and postnatal timepoint was good (Cronbach’s *α* = 0.72 to 0.80). The PHQ-4 variables were right-skewed, with most participants reporting fewer symptoms (see Statistical Analysis for how this was handled).

#### Child neurodevelopment

Neurodevelopment at 24 months was assessed using the Ages and Stages Questionnaire, Third Edition (ASQ-3), a reliable and valid screening tool evaluating communication, fine motor, gross motor, problem-solving, and personal-social skills (Squires et al. 2009). Total scores ranging from 0–60 were used. We previously found that children exposed to the pandemic at 24 months had lower personal-social skills compared to non-exposed children, and those exposed for longer periods had worse gross motor skills (Finegold et al. 2023). Thus, the present study focused on these specific outcomes.

#### Covariates

Regression and mediation analyses controlled for child age, maternal age at birth, child sex (0 = male, 1 = female), gestational age at birth (in weeks), birth weight, maternal race (0 = White, 1 = Non-White), maternal country of birth (0 = Canada, 1 = Outside Canada), maternal education (0 = Less than Bachelor’s degree, 1 = Bachelor’s degree or higher), and annual household income (0 = $150,000 CAD or less, 1 = above $150,000). Cutoffs were determined based on the distributions of responses to ensure roughly equal group sizes. These covariates are consistent with those used previously (Finegold et al. 2023) and were selected based on their known association with maternal mental health and child neurodevelopment (Guhn et al. 2020; Linsell et al. 2015).

### Statistical analyses

Analyses were conducted using R version 4.5.0 (R Project for Statistical Computing). To address missing data, the missRanger package was used to estimate missing values for covariates and PHQ-4 variables by employing a multivariate imputation algorithm based on random forests (Stekhoven & Bühlmann 2012). Preliminary analyses examined associations between pandemic exposure, maternal internalizing symptoms, and child neurodevelopment. Since the distribution of the PHQ-4 was non-normal, non-parametric tests were used. Wilcoxon rank-sum tests were used to test differences in maternal internalizing symptoms between pandemic-exposed and non-exposed mothers prenatally and at 8 and 24 months. Preliminary associations between maternal internalizing symptoms and child gross motor and personal-social skills were tested using Spearman’s correlations.

Next, negative binomial regression was used to examine associations between pandemic exposure and maternal internalizing symptoms, while linear regression was used to test associations between maternal internalizing symptoms and child outcomes. All regression tests adjusted for covariates. If both regression tests were at least marginally significant (*p* < 0.1), mediation analyses were used to test whether pandemic exposure was associated with child outcomes indirectly through maternal internalizing symptoms. The MAZE package in R was used for mediation analyses as it is uniquely designed to accommodate zero-inflated mediators (Jiang et al. 2023). The approach estimates the overall or natural indirect effect (NIE) and also decomposes it into two distinct components: one reflecting the continuous change in the mediator (NIE1), and the other reflecting the binary change from zero to non-zero (NIE2). To aid in model convergence and interpretability, all continuous covariates were standardized and mean-centered. Zero-inflated negative binomial (ZINB) and zero-inflated Poisson (ZIP) models were fitted for each mediator, and a final model was selected using the Akaike Information Criterion (AIC).

## Results

### Descriptive analyses

Table 1 presents the participant demographic characteristics, and Table 2 presents the distributions of the maternal internalizing symptoms and child outcome variables. At the prenatal assessment, 666 mothers were in the non-exposed group and 55 were in the prenatal-exposed group. At the 8-month assessment, 287 mothers were in the non-exposed group, 44 were in the pre/postnatally exposed group, and 110 were in the postnatal-only exposed group. At the final 24-month assessment, 247 mothers were in the non-exposed group, 55 were in the pre/postnatally exposed group, 212 were in the postnatal-only exposed group at 24 months only, and 110 were postnatal-only exposed at both 8 and 24 months (see Figure S1 for a study flowchart with sample sizes for the pandemic groups at each assessment timepoint).

**Table 1:** Demographic characteristics of sample (*N* = 838)

Variable	
Child sex, N (%)	
Male	426 (50.8)
Female	390 (46.5)
Missing (%)	2.6
Child age, mean (SD), months	2.2 (0.1)
Missing (%)	2.6
Gestational age, mean (SD), weeks	39.1 (1.5)
Missing (%)	2.6
Birthweight, mean (SD), grams	3291.0 (506.3)
Missing (%)	2.9
Maternal age, mean (SD), years	33.9 (3.8)
Missing (%)	3.0
Maternal race, N (%)	
White	529 (63.1)
Race other than White^a^	282 (33.7)
Missing (%)	3.2
Maternal birth country, N (%) Canada	589 (70.3)
Outside of Canada	225 (26.8)
Missing (%)	2.9
Maternal education, N (%) Bachelor’s or less	95 (11.3)
Graduate school	717 (85.6)
Missing (%)	3.1
Household income, N (%)	
≤ $150,000	302 (36.0)
> $150,000	481 (57.4)
Missing (%)	6.6

^a^ The racial groups included within the other or mixed category are South Asian, East or Southeast Asian, Middle Eastern, Black, Hispanic, Indigenous, and mixed race. White was chosen as the reference category since it was the most commonly endorsed by participants

**Table 2:** Descriptive statistics for ASQ-3 and PHQ-4 by pandemic exposure group

	Total Sample Median (IQR)	Non-Exposed Median (IQR)	Exposed Median (IQR)	
			Postnatal- Only Exposed	Pre/Postnatal-Exposed
Prenatal PHQ-4	1.0 (0.0–2.0)	1.0 (0.0–2.0)	-	1.5 (0.5–2.5)
12–16 weeks gestation	1.0 (0.0–2.0)			
28–32 weeks gestation	1.0 (0.0–2.0)			
8 month PHQ-4	1.0 (0.0–2.0)	1.0 (0.0–2.0)	1.0 (0.0–2.0)	1.0 (0.0–2.0)
24 month PHQ-4	1.0 (0.0–2.0)	1.0 (0.0–2.0)	1.0^†^ (0.0–2.0)	1.0 (0.0–2.0)
			2.0^‡^ (1.0–3.0)	-
ASQ-3 domains (24 months)				
Gross motor	55.0 (50.0–60.0)	60.0 (50.0–60.0)	55.0 (50.0–60.0)	
Personal-social	50.0 (45.0–60.0)	55.0 (50.0–60.0)	50.0 (45.0–56.25)	

Abbreviations: ASQ-3, Ages and Stages Questionnaire, Third Edition; PHQ-4,

Patient Health Questionnaire-4

The scale for the PHQ-4 is 0 (“not at all”) to 3 (“nearly every day”)

^†^24 month exposure only

^‡^8 and 24 month exposure

**Fig. 2:**
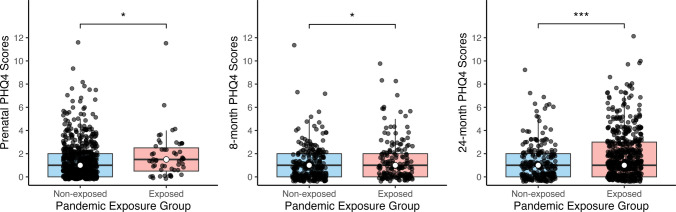
Differences in maternal mental health (PHQ-4) scores between pandemic-exposed and non-exposed mothers, as measured prenatally and at 8 and 24 months. Mothers with pandemic exposure had significantly more internalizing symptoms prenatally (*W* = 15,185.0, *p* = 0.03), at 8 months postnatal (*W* = 21,084.5, *p* = 0.04), and 24 months postnatal (*W* = 52,607.0, *p* < 0.001), compared to mothers with no pandemic exposure. In the figure, * *p* < 0.05; ** p < 0.01; *** *p* < 0.001

**Fig. 3:**
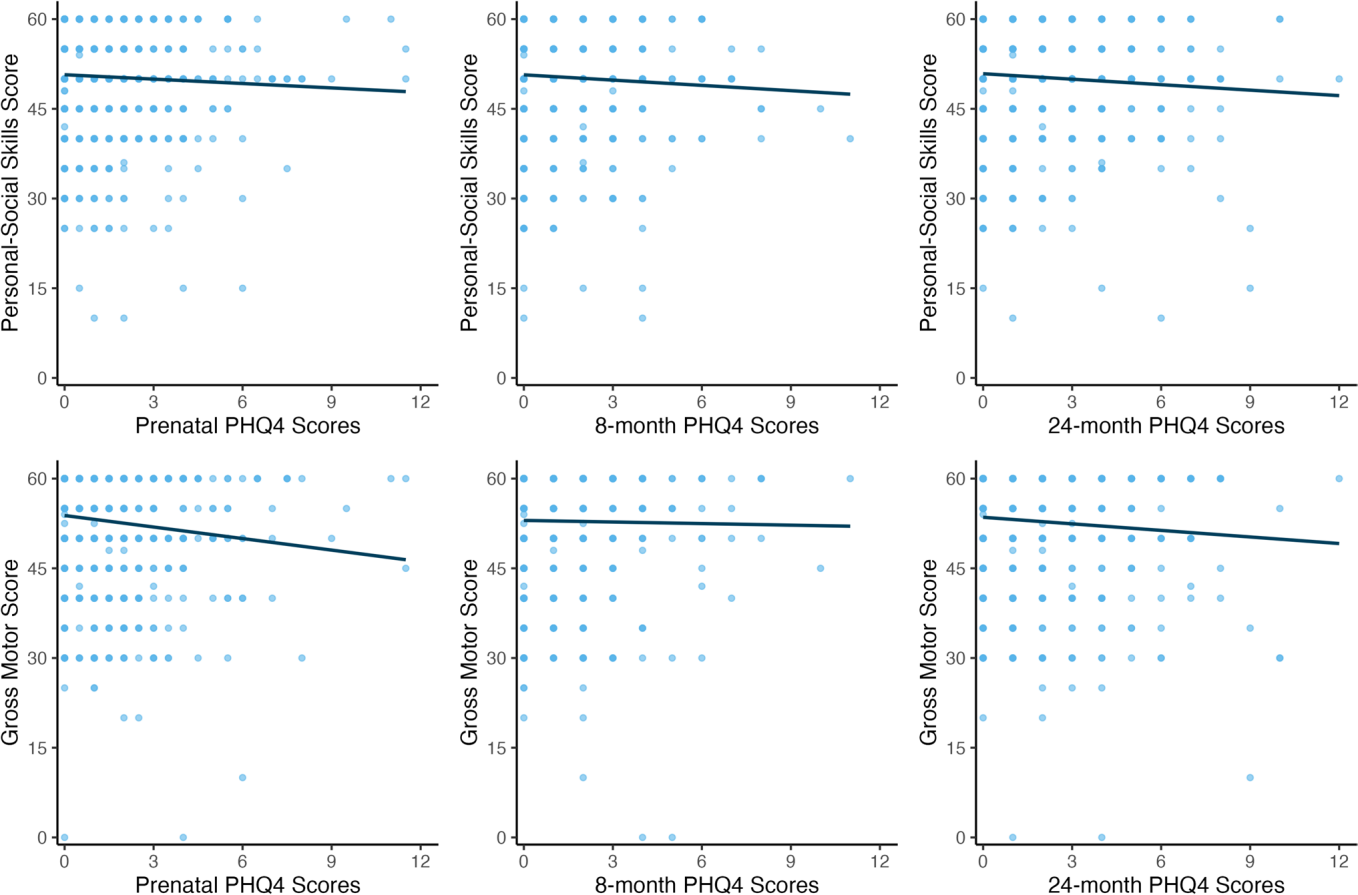
Association between maternal mental health symptoms and children’s gross motor and personal-social skills at 24 months. Children whose mothers reported higher internalizing during pregnancy had significantly lower gross motor (*ρ*(784) = −0.15, *p* < 0.001) and personal-social skills (*ρ*(780) = −0.07, *p* = 0.04) at 24 months

In unadjusted analyses, pandemic-exposed mothers had higher internalizing symptoms prenatally, at 8 months postnatally, and 24 months postnatally compared to mothers in the non- exposed group (Fig. 2). Irrespective of pandemic exposure, children whose mothers reported higher internalizing symptoms prenatally had significantly lower gross motor and personal-social skills at 24 months (Fig. 3). Maternal internalizing symptoms at 8 and 24 months were not significantly associated with child outcomes at 24 months.

### Pandemic exposure and maternal internalizing symptoms

Controlling for covariates, maternal internalizing symptoms were estimated to be 36% higher among mothers with prenatal pandemic exposure compared to mothers who were non-exposed prenatally (Table 3). At 8 months and 24 months, the expected rate of concurrent internalizing symptoms was significantly higher among postnatal-only exposed mothers compared to non-exposed mothers. The expected rate of internalizing symptoms was highest among mothers exposed to the pandemic at both 8 and 24 months, and higher than non-exposed mothers. Prenatal and postnatal exposure at both 8 and 24 months was marginally associated with lower rates of maternal internalizing symptoms relative to postnatal-only exposure at 8 and 24 months. However, in sensitivity analyses removing those assessed after May 5, 2023 (*N* = 32), this difference was no longer significant (see Table S1).

**Table 3:** Associations between pandemic exposure and maternal mental health symptoms, assessed using the PHQ-4

Predictor	Outcome	Rate Ratio (exp(* β*))	95% CI	*p*-value
Prenatal Assessment	Prenatal PHQ-4			
*Non-exposed prenatally vs. Exposed prenatally*		1.36	1.02, 1.80	0.03
8 month Assessment	8 month PHQ-4			
*Non-exposed vs. Postnatal-exposed only*		1.41	1.05, 1.88	0.03
*Non-exposed vs. Pre/postnatal-exposed*		1.34	0.87, 2.08	0.19
*Postnatal-exposed only vs. Pre/postnatal-exposed*		0.97	0.63, 1.50	0.89
24 month Assessment	24 month PHQ-4			
*Non-exposed vs. Postnatal-exposed only*		1.53	1.21, 1.94	< 0.001
*Non-exposed vs. Pre/postnatal-exposed*		1.24	0.86, 1.79	0.26
*Postnatal-exposed only vs. Pre/postnatal-exposed*		0.90	0.60, 1.34	0.60
8 and 24 month Assessment	24 month PHQ-4			
*Non-exposed vs. Postnatal-exposed only*		1.70	1.33, 2.17	< 0.001
*Postnatal-exposed only vs. Pre/postnatal-exposed*		0.73	0.52, 1.02	0.07

The results remained the same when analyzing the data with continuous variables for household income and maternal education

### Maternal internalizing symptoms and child neurodevelopment

Controlling for covariates and pandemic exposure, children whose mothers reported more internalizing symptoms during pregnancy had lower gross motor skills at 24 months (Table 4). Children whose mothers reported more internalizing symptoms at 8 months had marginally lower personal-social skills at 24 months. Finally, children whose mothers reported more internalizing symptoms at 24 months had marginally lower gross motor skills at 24 months. These results were largely replicated in analyses including categorical scores using standardized cut-offs (below the cut-off/at risk and above the cut off) for the ASQ-3 (see Table S3).

**Table 4:** Associations between maternal mental health symptoms, assessed using the PHQ-4, and children’s neurodevelopment at 24 months, assessed using domains from the ASQ-3 after controlling for pandemic exposure

Predictor	Outcome	N	Unstandardized B	Standardized β	95% Confidence Interval	*p*-value
	Gross Motor Skills					
Prenatal PHQ-4		719	−0.68	−0.12	−0.19, −0.04	**0.002**
8 month PHQ-4		450	0.02	0.003	−0.09, 0.10	0.94
24 month PHQ-4		800	−0.33	−0.07	−0.14, 0.00	**0.06**
	Personal-Social Skills					
Prenatal PHQ-4		717	−0.05	−0.008	−0.08, 0.06	0.82
8 month PHQ-4		448	−0.50	−0.09	−0.18, 0.00	**0.06**
24 month PHQ-4		796	−0.23	−0.05	−0.12, 0.02	0.17

The results remained the same when analyzing the data with continuous variables for household income and maternal education

### Maternal internalizing symptoms as a mediator of pandemic exposure

Only one indirect effect emerged as significant: prenatal maternal internalizing symptoms marginally mediated the association between prenatal pandemic exposure (versus non-exposure) and children’s gross motor skills at 24 months (NIE = −0.73 [−1.45, −0.01], *p* = 0.05). This effect was driven by the binary change from no maternal internalizing symptoms to any symptoms (NIE2 = −0.65 [−1.36, 0.06], *p* = 0.07) rather than the severity of symptoms. Thus, prenatal exposure to the pandemic may be associated with worse child gross motor skills at 24 months via the increased likelihood of any maternal internalizing symptoms during pregnancy. No other significant mediation effects were observed.

## Discussion

The current study examined associations between pandemic exposure, maternal internalizing symptoms, and child neurodevelopment at 24 months, as well as whether maternal internalizing symptoms mediated links between pandemic exposure and child outcomes. This is one of the first studies to longitudinally examine potential mediators of the association between pre- and postnatal pandemic exposure and children’s neurodevelopmental difficulties. At each pre- and postnatal timepoint, mothers assessed during the pandemic reported more concurrent internalizing symptoms compared to non-pandemic exposed mothers. Mothers exposed to the pandemic both prenatally and postnatally at both 8 and 24 months reported marginally lower internalizing symptoms than mothers exposed to the pandemic only postnatally at 8 and 24 months, though this effect did not hold in sensitivity analysis. Mothers with prolonged postnatal exposure (i.e., at both 8 and 24 months) reported the highest levels of internalizing symptoms. This suggests that mothers with extended postnatal pandemic exposure may have been particularly susceptible to worse mental health. However, these findings should be interpreted with caution due to the small group sizes, which may limit statistical power and reduce reliability of results.

Furthermore, higher maternal internalizing symptoms prenatally and at 24 months were related to poorer child gross motor development at 24 months, whereas children whose mothers had more internalizing symptoms at 8 months had marginally worse personal-social skills at 24 months. Finally, part of the negative association between prenatal pandemic exposure and children’s gross motor skills at 24 months operated indirectly through the presence of any maternal prenatal internalizing symptoms, rather than the extent of maternal internalizing symptoms. Our study is one of very few to longitudinally examine both pre- and postnatal pathways that may explain associations between pandemic exposure and early neurodevelopmental challenges. Our finding that prenatal, but not postnatal, maternal internalizing symptoms mediated the relation between pandemic exposure and children’s gross motor skills suggests that the prenatal window may be a period of increased vulnerability during times of collective stress and disruption.

Overall, the present study’s findings underscore the extent to which the COVID pandemic was associated with alterations in mothers’ mental health and well-being. While causal conclusions cannot be drawn, the natural experiment design provides strong support for the supposition that mothers who were pregnant or raising young children during the pandemic experienced more symptoms of anxiety and depression than women assessed before the pandemic. These results are consistent with epidemiological studies demonstrating increases in maternal distress, anxiety, and depression during the pandemic (Hessami et al. 2022; Racine et al. 2021). During early childhood, pandemic-related school and daycare closures and loss of childcare may have resulted in additional caretaking responsibilities that exacerbated pre-existing gender inequities (Andrade et al. 2022). Indeed, mothers reported both more pandemic stressors and increased mental health problems compared to fathers during the pandemic (Wade et al. 2021). During a period when mothers were experiencing high levels of work-from-home burnout (Delaney et al. 2023), they also had limited access to social supports (Grumi et al. 2021; Hetherington et al. 2018; Racine et al. 2019), an essential protective factor for mothers of young children (Hetherington et al. 2018; Racine et al. 2019). Perhaps especially vulnerable were pregnant women who faced unique stressors during the pandemic, including fears about contracting and transmitting the virus to their child, vaccine hesitancy, significant disruptions to prenatal care, and heightened social isolation during a period where support and connection are crucial for well-being (Liu & Fisher 2022; Panahi et al. 2020).

Our finding that prenatal maternal internalizing symptoms was a marginal mediator of the association between prenatal pandemic exposure and children’s gross motor skills at 24 months is consistent with prior work on the neurodevelopmental impact of prenatal stress during natural disasters and other crises (Martínez-González et al. 2023). Specifically, higher prenatal stress during weather-related natural disasters is associated with lower motor abilities in infants and young children (Cao et al. 2014; Moss et al. 2017, 2018). During the pandemic specifically, it has been shown that prenatal stress and anxiety symptoms are related to more difficulties with communication, fine motor, social-emotional, and attentional in infants (Liu et al. 2024; López-Morales et al. 2023; Werchan et al. 2024). Papadopoulous et al. (Papadopoulos et al. 2022) found that prenatal stress and depression during the pandemic predicted lower motor skills during the first 2 months of life. Emerging research demonstrates that areas in the brain responsible for motor function, such as the cerebellum, may be particularly sensitive to prenatal stress (Koning et al. 2017). The cerebellum is highly susceptible to environmental stressors due to its rapid development during late pregnancy and continued development through the second year of life (Limperopoulos et al. 2005; Volpe 2009), with a high concentration of glucocorticoid receptors relative to other brain regions (Bennett et al. 2017; Pavlík & Buresová, 1984). Increases in prenatal stress have been linked with alterations in the structure and function of the cerebellum and later motor delays and cognitive difficulties in offspring in both animal studies and studies of early adversity (Bauer et al. 2009; Bennett et al. 2017; Patin et al. 2004; Ulupinar & Yucel 2005).

To our knowledge, the current study is the first to demonstrate that prenatal maternal internalizing symptoms during the pandemic are a risk factor for worse neurodevelopment through the second year of life and may be a mechanism linking prenatal pandemic exposure to children’s neurodevelopment. Our finding that the presence of any prenatal maternal internalizing symptoms partially mediated the link between prenatal pandemic exposure and children’s gross motor development suggests that stress during pregnancy may have posed a particular threat to children’s development during the pandemic. Notably, the mere presence of prenatal maternal internalizing symptoms— rather than greater symptom severity—accounted for the relation between prenatal pandemic exposure and children’s neurodevelopment, indicating that even relatively low levels of maternal psychological distress during times of ecological crisis may be consequential for children’s outcomes. This finding may be of particular relevance to public health officials and health care providers developing interventions during future pandemics or disasters. Further, our results are supported by developmental theories such as the Developmental Origins of Health and Disease (DOHaD) framework and the prenatal programming hypothesis (Barker 2004), which together posit that the prenatal brain, which undergoes rapid growth and is highly malleable, is especially vulnerable to environmental factors, including maternal stress (Lautarescu et al. 2020; Monk et al. 2019; Van den Bergh et al. 2020). Prenatal maternal stress is a significant risk factor for later psychopathology and neurodevelopmental disorders (Talge et al. 2007; Van den Bergh et al. 2020), and a number of candidate pathways have been suggested for how maternal stress alters fetal development and impacts later developmental outcomes. Proposed mechanisms include alterations in the regulation of the fetus’s hypothalamic–pituitary–adrenal (HPA) axis, epigenetic modifications affecting the transcription of glucocorticoid-related genes in the placenta, and changes to fetal immune function via the transfer of maternal cytokines through the placenta (Lautarescu et al. 2020; Monk et al. 2019; Van den Bergh et al. 2020). Our findings align with the DOHaD framework, suggesting that prenatal maternal distress during times of ecological crises may become biologically embedded through prenatal programming processes, with small but measurable implications for early neurodevelopment.

Strengths of the current study included the longitudinal design and measurement of maternal internalizing symptoms both pre- and postnatally, enabling an examination of timing effects on maternal mental health and child outcomes. Additionally, the natural experiment design and rigorous control for confounding variables are features that are rare in pandemic research involving mothers and children. Limitations included the relatively sociodemographically advantaged sample, which may limit generalizability. However, considering that the study sample was relatively sociodemographically advantaged, and given that SES may confer some protections against stress and disruption caused by the pandemic, it may be that our findings underestimate the true size of effects of the pandemic in the population. Furthermore, mothers reported on their own mental health and child’s development, potentially introducing shared rater bias. Importantly, the ASQ-3 and PHQ-4 are screeners for child development and internalizing symptoms, respectively, and are not diagnostic tests. Moreover, data about other potential risk or protective factors (e.g., the presence and quality of siblings, partner engagement) that may have moderated the observed associations were not available for inclusion in this study. Lastly, due to small sample sizes in certain exposure groups, analyses may have been underpowered to detect some associations. Despite these limitations, the present study’s findings offer valuable insight into how pandemic-related disruptions may have shaped maternal and child wellbeing, underscoring the importance of continued support for families during and after public health emergencies.

## Supplementary Information

Below is the link to the electronic supplementary material.

Supplementary file1 (DOCX 30 KB)

## Data Availability

No datasets were generated or analysed during the current study.
