# Supplementary material for: Maternal internalizing symptoms as a mechanism linking pre- and postnatal COVID-19 pandemic exposure with preschool-aged children’s neurodevelopment
Source: Arch Womens Ment Health. 2026 Mar 26;29(2):55. doi: 10.1007/s00737-026-01686-2 (PMC13021730; doi:10.1007/s00737-026-01686-2)
Supplement: Supplementary file 1 — Supplementary file1 (DOCX 30 KB) [file 737_2026_1686_MOESM1_ESM.docx]

**Supplementary Materials**

**Supplemental Results**

Sensitivity analyses were conducted that excluded the 24-month assessment data of 32 participants who were assessed after the WHO declared the end of the pandemic on May 5, 2023. These participants completed their 24-month assessment after May 5, 2023 through the end of the present study on February 1, 2024. In the main text, these participants are part of the *pre/postnatal-exposed group* because they were exposed to the pandemic prenatally and for the majority of their postnatal life. For the sensitivity analyses, regression tests that included the *pre/postnatal exposed group* as one of the pandemic exposure groups and/or outcome measures at 24 months (e.g., the PHQ-4 at 24 months or ASQ-3 domains) were re-run excluding the 32 participants whose 24-month assessments were after May 5, 2023. The overall pattern of results remained consistent, with the primary difference being a now non-significant difference in maternal PHQ-4 scores between those exposed to the pandemic only postnatally at 8 and 24 months versus those exposed prenatally and postnatally at 8 and 24 months, compared to the results in the main text. The smaller pre/postnatal group in this sensitivity analysis likely contributed to the much wider 95% CI and non-significant effect in this case. The results of these tests are presented below.

**Table S1.** Associations between pandemic exposure and maternal mental health symptoms, assessed using the PHQ-4.

| **Predictor** | **Outcome** | **Rate Ratio (exp(*β***) | **95% CI** | ***p*-value** |
| --- | --- | --- | --- | --- |
| **24 month Assessment** | 24 month PHQ-4 |  |  |  |
| *Non-exposed vs.*  *Pre/postnatal-*  *exposed* |  | 1.51 | 0.91, 2.53 | 0.11 |
| *Postnatal-exposed*  *only vs. Pre/postnatal-*  *exposed* |  | 1.06 | 0.64, 1.74 | 0.83 |
| **8 and 24 month Assessment** | 24 month PHQ-4 |  |  |  |
| *Postnatal-exposed only*  *vs. Pre/postnatal-exposed* |  | 0.97 | 0.63, 1.48 | 0.90 |

**Table S2.** Associations between maternal mental health symptoms, assessed using the PHQ-4, and children’s neurodevelopment at 24 months, assessed using domains from the ASQ-3 after controlling for pandemic exposure.

| **Predictor** | **Outcome** | **Unstandardized B** | **Standardized β** | **95% CI** | ***p*-value** |
| --- | --- | --- | --- | --- | --- |
| 24-month PHQ-4 | Gross Motor Skills | -0.34 | -0.07 | -0.14, 0.01 | 0.07 |
| 24-month PHQ-4 | Personal-Social Skills | -0.27 | -0.06 | -0.13, 0.01 | 0.11 |
|  |  |  |  |  |  |
|  |  |  |  |  |  |

Sensitivity analyses were conducted to test the preliminary regression results and mediation model when the ASQ-3 domains were represented as binary variables. Participants were either in the typically developing range for personal-social and gross motor skills if their score was above the standard cut-off, or in the ‘at risk’ range if their score fell below the standard cut-off. Results of logistic regression models using cut-off scores for the ASQ-3 were largely consistent with the results of linear regression models using continuous scores. The association between maternal internalizing symptoms at 24 months and children’s personal-social skills was marginally significant (*p*- value = 0.06) when continuous scores for the ASQ-3 were used, but not significant when using cut-off scores. The mediation effect remained marginally significant.

**Table S3.** Associations between prenatal maternal internalizing symptoms, assessed using the PHQ-4, and risk of neurodevelopmental difficulties, assessed using the ASQ-3.

| Predictor | Outcome | Odds Ratio (95% Confidence Interval) | *p*-value |
| --- | --- | --- | --- |
|  | Gross Motor Skills |  |  |
| Prenatal PHQ-4 |  | 0.75 (0.58, 1.01) | **0.03** |
| 8 month PHQ-4 |  | 0.88 (0.49, 2.19) | 0.72 |
| 24 month PHQ-4 |  | 0.67 (0.53, 0.84) | **<0.001** |
|  | Personal-Social Skills |  |  |
| Prenatal PHQ-4 |  | 1.12 (0.85, 1.63) | 0.48 |
| 8 month PHQ-4 |  | 1.09 (0.76, 1.78) | 0.70 |
| 24 months PHQ-4 |  | 0.92 (0.76, 1.15) | 0.41 |

**Figure S1.** Study flowchart depicting sample sizes in the pandemic groups at each assessment timepoint (e.g., prenatal, 8-month, and 24-month assessments).

Prenatal Assessment

 Non-exposed to the

pandemic: 666

 Prenatal-exposed: 55

8-Month Assessment

 Non-exposed to the pandemic: 287

 Pre/postnatal exposed: 44

 Postnatal-only exposed: 110

24-Month Assessment

 Non-exposed to the pandemic: 247

 Pre/postnatal exposed: 55

Postnatal-only exposed at 24-months: 212

 Postnatal only exposed at 8- and 24-months: 110
